# Allometric growth in the extant coelacanth lung during ontogenetic development

**DOI:** 10.1038/ncomms9222

**Published:** 2015-09-15

**Authors:** Camila Cupello, Paulo M. Brito, Marc Herbin, François J Meunier, Philippe Janvier, Hugo Dutel, Gaël Clément

**Affiliations:** 1 Departamento de Zoologia, Universidade do Estado do Rio de Janeiro, R. São Francisco Xavier, 524-Maracanã, Rio de Janeiro 20550–900, Brazil; 2 Département Écologie et Gestion de la Biodiversité, UMR 7179 CNRS–MNHN, Mécanismes adaptatifs des organismes aux communautés, Muséum national d'Histoire naturelle, 57 rue Cuvier, Paris 75231, France; 3 Département des Milieux et Peuplements Aquatiques, UMR 7208 (CNRS–IRD–MNHN–UPMC), Equipe BOREA, Muséum national d'Histoire naturelle, CP026, 43 rue Cuvier, Paris 75231, France; 4 Centre de Recherche sur la Paléobiodiversité et les Paléoenvironnements (CR2P, UMR 7207), Sorbonne Universités, MNHN, CNRS, UPMC-Paris6, Muséum national d'histoire naturelle, 57 rue Cuvier, CP38, Paris F-75005, France; 5 Laboratory for Evolutionary Morphology, RIKEN, 2-2-3 Minatojima-minami, Chuo-ku, Kobe 650-0047, Japan

## Abstract

Coelacanths are lobe-finned fishes known from the Devonian to Recent that were long considered extinct, until the discovery of two living species in deep marine waters of the Mozambique Channel and Sulawesi. Despite extensive studies, the pulmonary system of extant coelacanths has not been fully investigated. Here we confirm the presence of a lung and discuss its allometric growth in *Latimeria chalumnae*, based on a unique ontogenetic series. Our results demonstrate the presence of a potentially functional, well-developed lung in the earliest known coelacanth embryo, and its arrested growth at later ontogenetic stages, when the lung is clearly vestigial. The parallel development of a fatty organ for buoyancy control suggests a unique adaptation to deep-water environments. Furthermore, we provide the first evidence for the presence of small, hard, flexible plates around the lung in *L. chalumnae*, and consider them homologous to the plates of the ‘calcified lung' of fossil coelacanths.

## Abstract (web summary)

The presence of a pulmonary system in fossil coelacanths has only recently been identified, with little known about homologues in living species. Here, Cupello *et al.* confirm the presence of a lung in the extant species *Latimeria chalumnae* and report its growth during different stages of development.

## Graphical abstract

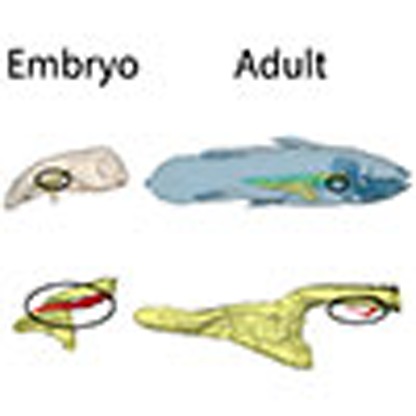

Coelacanths form a clade of predatory sarcopterygian fishes known from the early Devonian (−410 Myr) to Recent, with the iconic species *Latimeria chalumnae*^1^ and *L. menadoensis*^2^,^3^. Fossil coelacanths are morphologically and taxonomically diverse (with more than 130 species), and globally distributed in various aquatic environments (fresh, brackish to marine waters)^4^. The evolutionary history of coelacanths is long and complex, and the living coelacanths cannot be considered as living fossils. There is a large gap of ∼70 Myr between the extant *Latimeria* and the youngest fossil coelacanth remains, *Megalocoelacanthus dobei* and an isolated angular of a mawsoniid coelacanth, both from the Upper Cretaceous^5^,^6^,^7^. This absence from the Cenozoic fossil record caused this group to be long considered as extinct since the Cretaceous/Palaeogene (K/Pg) environmental crisis. The discovery in 1938 of the extant species *L. chalumnae* offshore South Africa in the Indian Ocean^1^ was followed by numerous articles on the anatomy, physiology and phylogeny of this group, turning it into either an iconic ‘living fossil' or a ‘Lazarus taxon'; that is, a born-again member of a reputedly extinct group^1^,^4^,^8^,^9^,^10^,^11^.

*Latimeria* is a heavily built fish living in rocky environments between 110 and 400 m deep in the coastal waters of the Mozambique Channel and of Sulawesi^12^,^13^,^14^. This large animal (up to 2 m long) is ovoviviparous^15^. The young develop in the oviduct of the female, which can give birth to 26 live pups of about 35 cm long^16^. Juvenile coelacanths (below 80 cm long) are rarely observed or caught^17^.

The presence of a large calcified sheath in the abdominal cavity of fossil coelacanths has been known since the 19th century^18^,^19^ but was previously regarded as either an ‘internal osseous viscus' (unknown internal organ), a bladder or swimbladder. Only recently this organ has been formally described in Palaeozoic and Mesozoic coelacanths as a pulmonary organ composed of large and rounded calcified plates, positioned ventrally relative to the gut, and with a single anterior opening under the opercle^20^.

Although the anatomy of living coelacanth has been extensively studied, little is known about its lung. *L. chalumnae* does not possess a calcified organ in its abdominal cavity, but a short oesophageal diverticulum surrounded by a fatty organ. Many aspects of the anatomy and the development of these structures in *Latimeria* remain unclear, as well as their homology to the calcified organ of many fossil coelacanths.

Here we confirm the presence of a lung in *L. chalumnae* and present its allometric growth based on a unique ontogenetic series. We also propose the first evidence of the homology between this lung and the ‘calcified lung' of fossil coelacanths, based on the presence of small plates around the lung in the extant species. The parallel development of a fatty organ for buoyancy control suggests a unique adaptation to deep water.

## Results

### The lung of *Latimeria*

**Figure 1:**
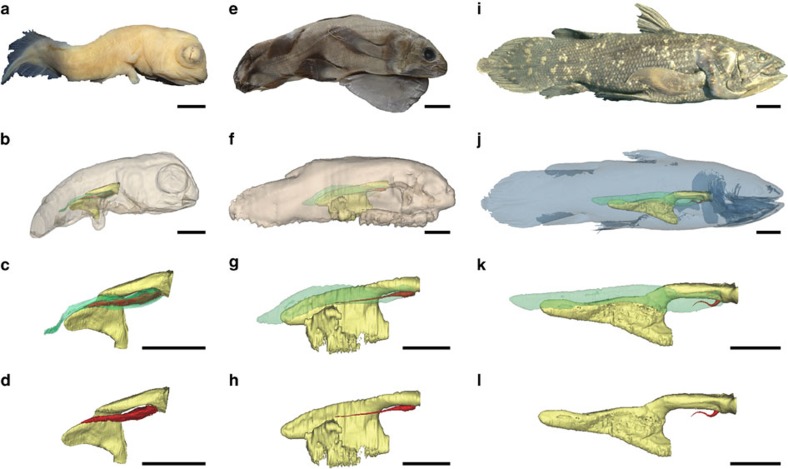
Three-dimensional reconstructions of the pulmonary complex of *L. chalumnae* at different ontogenetic stages. Right lateral views of the specimens showing the allometric growth of the lung. (**a**–**d**) Early embryo CCC 202.1 (4 cm TL). (**e–h**) Embryo with yolk sac CCC 29.5 (32.3 cm TL). (**i**–**l**) Adult CCC 22 (130 cm TL). Yellow, oesophagus and stomach; green, fatty organ; red, lung. Scale bar, 0.5 cm (**a**–**d**); 2.5 cm (**e**–**h**); 10 cm (**i**–**l**).

**Figure 2:**
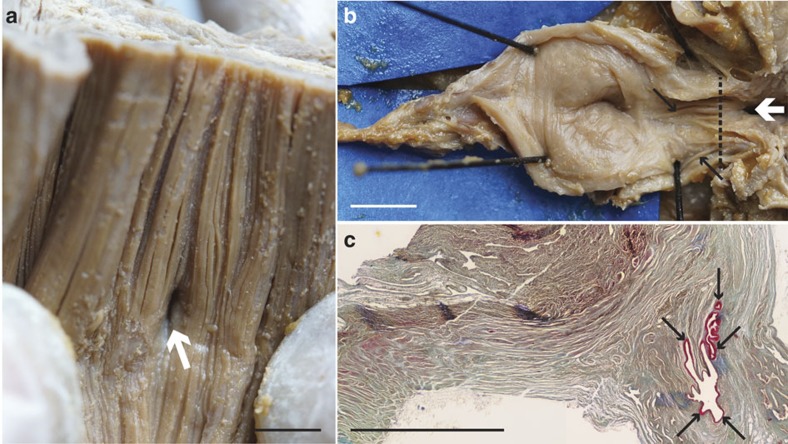
Evidence of pulmonary complex in *L. chalumnae*. (**a**) Internal view of the ventral wall of the oesophagus showing the non-obliterated opening between oesophagus and lung in *L. chalumnae* CCC 3. Dorsal view of the oesophagus (anterior to the top). (**b**) Anterior part of the vestigial lung lumen from the dissection of the adult specimen CCC 3, ventral view, anterior to the right. (**c**) Histological thin section of the vestigial lung of CCC 5 in the region of the black dashed line in **b**. White arrows indicate the non-obliterated opening in specimen CCC 3, and black arrows indicate the invaginations of the internal wall of *L. chalumnae* vestigial lung in specimens CCC 3 and CCC 5. Scale bar, 0.5 cm (**a**–**c**).

The pulmonary complex of *L. chalumnae* comprises a vestigial lung (oesophageal diverticulum) derived from the ventral portion of the oesophagus (Fig. 1). The presence of an oesophageal diverticulum in a medio-ventral position relative to the oesophagus, its non-obliterated opening (Fig. 2a), its internal cavity (Fig. 2b), as well as invaginations in the anterior part of its internal wall (Fig. 2b,c) characterizes this structure as a lung.

The vestigial lung of *L. chalumnae* is included in the anteriormost part of a large, closed and tubular sheath filled with fat (Fig. 1). This so-called ‘fatty organ'^10^,^20^, previously referred in the literature as a swimbladder^21^, air bladder^4^, fatty lung^20^ and modified lung or bladder^22^, has a ventral position relative to the oesophagus, turns dorsally relative to the stomach and reaches the posterior wall of the abdominal cavity^10^. This organ in *Latimeria* is structurally different from the swimbladder of many actinopterygians that is, in the latter group, an air sac with, or without, an anterior opening located on the dorsal side of the oesophagus.

**Figure 3:**
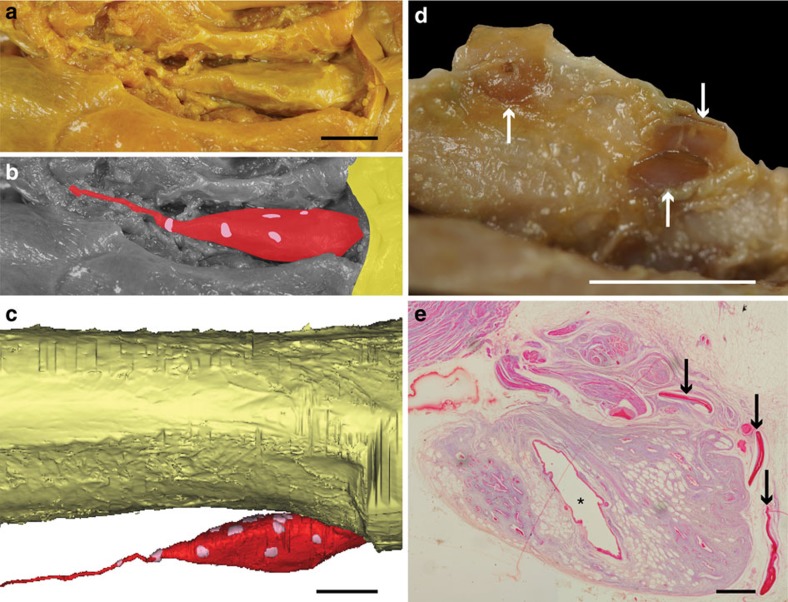
**Small, hard but flexible plates surrounding the vestigial lung of adult specimens of** ***L. chalumnae***. (**a**,**b**) Ventral view of the vestigial lung of CCC 28, anterior to the right (yellow, oesophagus; red, vestigial lung; pink, hard plates). (**c**) Right lateral view of the three-dimensional reconstruction of the oesophagus, the vestigial lung and hard plates of CCC 28 (yellow, oesophagus; red, vestigial lung; pink, hard plates). (**d**) Close-up on three hard plates (arrows) in the sheath that surrounds the vestigial lung of CCC 3. (**e**) Histological thin section (azocarmine coloration) of the vestigial lung of CCC 5; arrows indicating the hard plates and asterisk pointing to the lumen of the vestigial lung. Scale bars, 1 cm (**a**–**c**); 0.5 cm (**d**); 0.1 cm (**e**).

Dissections, three-dimensional reconstructions and histological analyses have revealed small, hard, but flexible plates scattered around the vestigial lung in adult specimens but not around the fatty organ (Fig. 3a–e). These plates correspond to the misinterpreted ‘*artères involuées*'^10^, and are quite similar in shape and position to the bony plates found in many fossil coelacanth taxa^20^.

### The lung development in *Latimeria*

There is a marked allometric growth of the lung compared with the growth of the fatty organ (Fig. 1). While the fatty organ's proportions relative to the total length (TL) increase proportionally according to the ontogenetic stages of individuals, the relative proportions between the lung and the TL of the specimens decrease in the juvenile (Supplementary Fig. 1) before stabilizing in the adult stage (Fig. 1i–l).

Moreover, the ratio between lung's length and the fatty organ's length is much higher in embryos (Fig. 1a–h) than in the juvenile (Supplementary Fig. 1) and adult (Fig. 1i–l) specimens. In the earliest embryo CCC 202.1 (4 cm TL), the lung's length corresponds to more than half of the length of the fatty organ, whereas this ratio decreases considerably in the juvenile specimen (Supplementary Fig. 1).

The oesophageal diverticulum of the earliest embryo is inflated throughout its length, which likely foreshadows a normal developmental stage towards a functional organ (Fig. 1d). In the following successive ontogenetic stages (CCC 29.5, CCC 162.21) the oesophageal diverticulum is markedly reduced to become a very thin and long filament here called the ‘residual filament', the inflated part being restricted to the anteriormost region (Fig. 1h; Supplementary Fig. 2).

## Discussion

Our discovery of hard but flexible plates around the vestigial lung (oesophageal diverticulum) of adult specimens of *L. chalumnae* documents the first evidence of the homology between its lung and the long debated ‘calcified lung' of fossil coelacanths (Supplementary Figs 3 and 4), questioning previous hypothesis of the fatty organ as part of the pulmonary complex^10^. In the Cretaceous coelacanth *Axelrodichthys*, the ossified plates surrounding the lung most probably had a function in lung volume regulation, with the plates moving over each other to accommodate volumetric changes^20^, while in *L. chalumnae* these plates are a rudimentary anatomical structure.

Here we demonstrate also the presence of a well-developed, potentially functional, lung in the earliest known *L. chalumnae* embryo, suggesting the presence of a functional lung in early fossil coelacanths. These animals lived in shallow brackish, fresh or marine environments and, like in some extant lungfishes and polypteriforms, air breathing might have been an essential respiratory need. The presence of a lung in *Latimeria* confirms that this organ is a general character for all osteichthyans, as it is also present in basal actinopterygians, lungfishes and tetrapods, notably land vertebrates^23^,^24^.

The extant coelacanth *L. chalumnae* has no functional air-breathing organ^10^, and the main oxygen supply is provided through the gills^25^. However, the low surface area of the *Latimeria* gills and the thick tissue barrier separating water from blood in the gill filaments^26^ could be evidence for a low branchial oxygen intake capacity in fossil coelacanths, which required a supplementary air-breathing supply, especially in shallow-water hypoxic conditions. In the Mesozoic Era, adaptation of some coelacanths to deep marine water, an environment with very low variations of oxygen pressure, may have triggered the total loss of pulmonary respiration, the marked reduction of the lung and the increase of the fatty organ as a buoyancy adaptation to deeper environments.

This adaptation to deep water may help explain the survival of some coelacanths throughout the Cretaceous/Palaeogene (K/Pg) environmental crisis, whereas Late Cretaceous coelacanths inhabiting shallow waters disappeared. Therefore, the apparent absence of coelacanths in the fossil record since the end of the Mesozoic is merely due to a lack of Cenozoic fossil coelacanths so far, and scarce preservation of Cenozoic deep-water sediments. Although we cannot know whether the fatty organ ever existed in fossil forms, due to its unique soft-tissue constitution, this organ in *Latimeria* has a function in buoyancy control. Such an organ seems to be functionally analogous to the liver of sharks^27^,^28^, the fat-invested string of tissue of some deep-water ray-finned fishes^29^ or the spermaceti of some cetaceans^30^,^31^.

## Methods

### Specimen information

The five different ontogenetic stages of *L. chalumnae* imaged using X-ray tomography come from Tanzania, Comoro Islands and Mozambique: CCC 202.1 (SAIAB 76199) is an early embryo of 4 cm TL, discovered inside the female CCC 202 fished in Tanzania in 2005. CCC 29.5 (MNHN 26.5) is a late embryo with a yolk sac, 32.3 cm TL, discovered inside the female CCC 29, fished in Comoro Islands in 1962. CCC 162.21 (ZSM 28409) is a late embryo without a yolk sac, 35.6 cm TL, discovered inside the female CCC 126 fished in Mozambique in 1991. CCC 94 (MNHN C79) is a young female, 42.5 cm TL, fished in Comoro Islands in 1974. The adult specimens are the following: CCC 22 (MNHN C20), male, 130 cm TL, Comoro Islands, 1960. CCC 28 (MNHN C25), male, 130 cm TL, Comoro Islands, 1962 (isolated viscus). Direct anatomical observations were made from new dissections of specimens CCC 28 and CCC 3 (MNHN C3, isolated viscus from an adult male of 129 cm TL, fished in Comoro Islands in 1953). Histological observations were made from unpublished thin sections prepared from specimen CCC 5 (MNHN C5, adult male of 127 cm TL, Comoro Islands, 1954) by Millot, Anthony and Robineau. More details on the information inventory of these specimens are provided elsewhere^17^,^32^. The histological material is deposited in the Collection of Comparative Anatomy of the Muséum national d'Histoire naturelle (France). The fossil adult specimen of *Macropoma mantelli* Agassiz, 1843 NHMUK PV P 2051, was recovered from Cretaceous Chalk Formation, Sussex (England).

Institutional abbreviations: SAIAB, South African Institute for Aquatic Biodiversity, Grahamstown (South Africa); MNHN, Muséum national d'Histoire naturelle; ZSM, Zoologische Staatssammlung, München (Germany); NHMUK, Natural History Museum United Kingdom, London (UK). CCC, Coelacanth Conservation Council.

### X-ray tomography

The specimens CCC 202.1, CCC 29.5, CCC 162.21 and CCC 94 were scanned using long propagation phase-contrast synchrotron X-ray microtomography at the ID19 beamline of the European Synchrotron Radiation Facility, Grenoble (France). CCC 202.1 was imaged supported in a glass cylinder vase filled with ethanol (resulting in a negative contrast agent effect), voxel size 6.5 μm, with a high-quality pink beam using the ID19 W150 wiggler at a gap of 50 mm filtered by 2 mm of aluminium, 0.25 mm of copper and 0.2 mm of gold. The scintillator was a 250-μm-thick LuAG:Ce (lutetium–aluminium–garnet) crystal. The resulting detected spectrum was then centred on 77 keV, with a bandwidth of 17 keV FWHM (full width at half maximum). The detector was a FreLoN 2K charge coupled device (CCD) camera^33^ mounted on a lens system. To obtain a sufficient propagation phase-contrast effect, a distance of 3 m between the sample and the detector was used. The final reconstruction (13 μm) was obtained after binning. CCC 162.21 was scanned in a plastic tube filled with water, voxel size 30.45 μm, using the ID19 W150 wiggler at a gap of 50 mm filtered by 2 mm of aluminium, 0.25 mm of copper and 0.25 mm of tungsten. The scintillator, detector and distance between the sample and the detector were the same as for CCC 202.1. The final reconstruction (60.90 μm) was obtained after binning. Specimens CCC 29.5 and CCC 94 were scanned in a plastic tube filled with water, voxel size 28.43 μm, using a propagation distance of 13 m to maximize the phase-contrast effect. For CCC 94, the beam produced by the wiggler at a gap of 30 mm was filtered by 2 mm of aluminium and 15 mm of copper, resulting in an average detected energy of 170 keV with a bandwidth of 85 keV FWHM. For CCC 29.5, a lower energy was used: wiggler at a gap of 45 mm filtered by 2 mm of aluminium and 6 mm of copper. The resulting spectrum had an average detected energy of 120 keV with a bandwidth of 57 keV FWHM. For both specimens the detector camera was a FreLoN 2K charge coupled device^33^ mounted on a lens system composed of a 750-μm-thick LuAG:Ce scintillator. The final reconstructions (113.72 μm for CCC 29.5 and 85.29 μm for CCC 94) were obtained after binning. In all cases, the slices were reconstructed using a filtered back-projection algorithm coupled with a single distance phase-retrieval process^34^,^35^. For each sample, all the sub-scans were reconstructed separately, converted into 16-bit TIFF stacks and then concatenated to generate a single complete scan of each specimen. The ring artefacts were corrected on the reconstructed slices using a specific tool developed at the European Synchrotron Radiation Facility^36^. A high-resolution computerized axial tomography scanning (CAT scan) was performed for the specimen CCC 22 in a Parisian Hospital (France) using the following scanning parameters: effective energy 120 kV, current 158 mA, voxel size 742 μm and 1,807 views. For the specimen CCC 28, a CAT scan was acquired at the Platform AST-RX of the Muséum national d'Histoire naturelle, using an effective energy of 245 kV, current 430 mA, voxel size 54.24 μm, and 2,550 views. Images were reconstructed and exported into 16-bit TIFF stacks using the phoenix datos|x 2.0 reconstruction software, and exported into 16-bit TIFF stacks. For all the specimens, segmentation and three-dimensional rendering were realized at the Palaeontology Imaging Unit of the MNHN Département Histoire de la Terre/UMR 7207 CR2P CNRS/MNHN/UPMC using the software MIMICS Innovation Suite 16.0 (Materialise).

## Additional information

**How to cite this article:** Cupello, C. *et al.* Allometric growth in the extant coelacanth lung during ontogenetic development. *Nat. Commun.* 6:8222 doi: 10.1038/ncomms9222 (2015).

## Supplementary Material

Supplementary Information Supplementary Figures 1-4
