# Supplementary material for: Allometric growth in the extant coelacanth lung during ontogenetic development
Source: Nat Commun. 2015 Sep 15;6:8222. doi: 10.1038/ncomms9222 (PMC4647851; doi:10.1038/ncomms9222)
Supplement: Supplementary Information — Supplementary Figures 1-4 [file ncomms9222-s1.pdf]

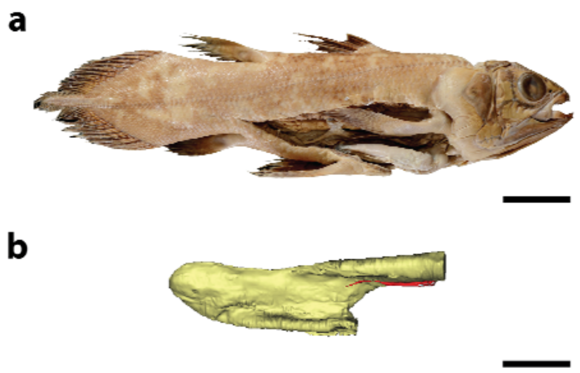

Supplementary figure 1 | Three-dimensional reconstruction of the vestigial lung in a juvenile *Latimeria chalumnae*. a, photograph of specimen CCC 94 (42,5 cm TL) in right lateral view. **b,** right lateral view of the three-dimensional reconstruction showing the lung, oesophagus and stomach. Yellow, oesophagus and stomach; red: vestigial lung. Scale bars: **a,** 5 cm; **b,** 2.5 cm.

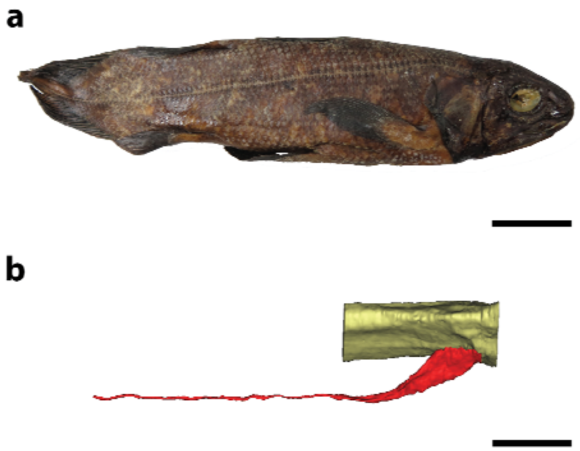

Supplementary figure 2 | Three-dimensional reconstruction of the vestigial lung in a late embryo without yolk sac of *Latimeria chalumnae*. a, mirror image of the left lateral view of the specimen CCC 162.21 (35.6 cm TL). **b,** right lateral view of the three-dimensional reconstruction, showing the lung with its residual filament. Yellow, oesophagus; red, vestigial lung. Scale bars: **a,** 5 cm; **b,** 1 cm.

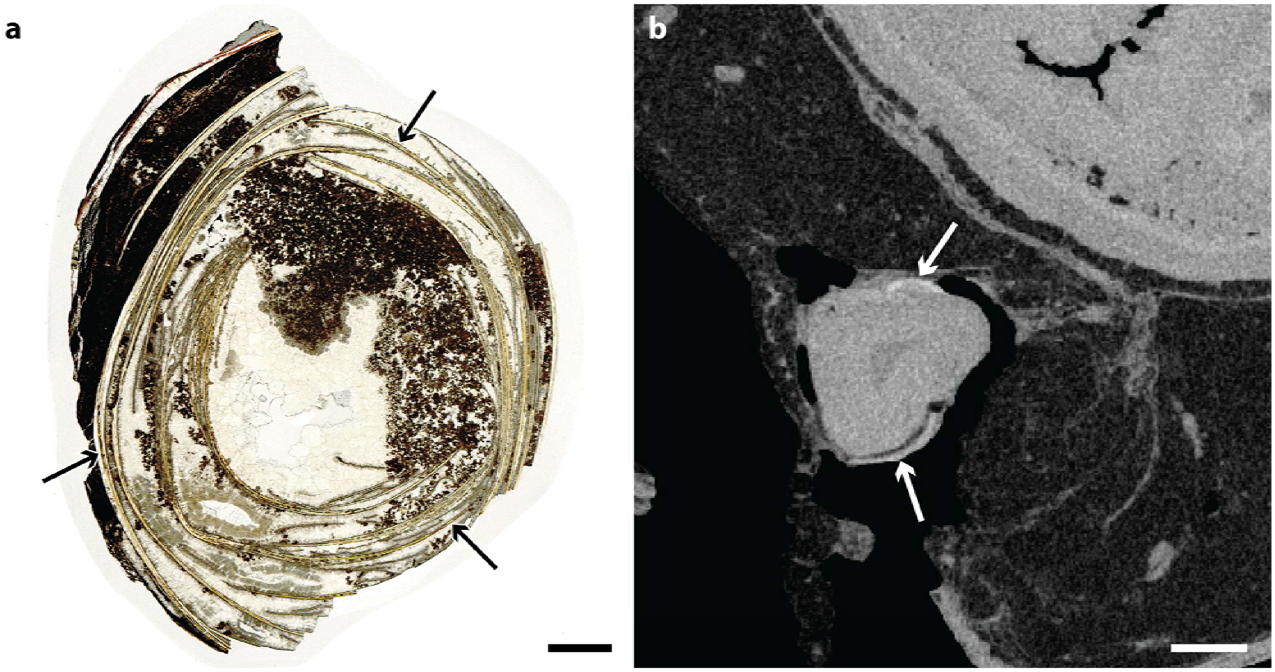

Supplementary figure 3 | Comparison between the calcified plates surrounding the lung of a fossil coelacanth and the hard but flexible plates of *Latimeria*. a, section of an uncrushed calcified bladder of the Cretaceous coelacanth *Axelrodichthys araripensis* from Brazil showing the calcified plates (arrows). **b,** section of a high-resolution computerized axial tomography scan of the vestigial lung (oesophageal diverticulum) of CCC 28 with two hard plates surrounding this organ (arrows). Oesophagus on top right corner. Scale bars: **a,** 0.5 cm; **b,** 0.25 cm.

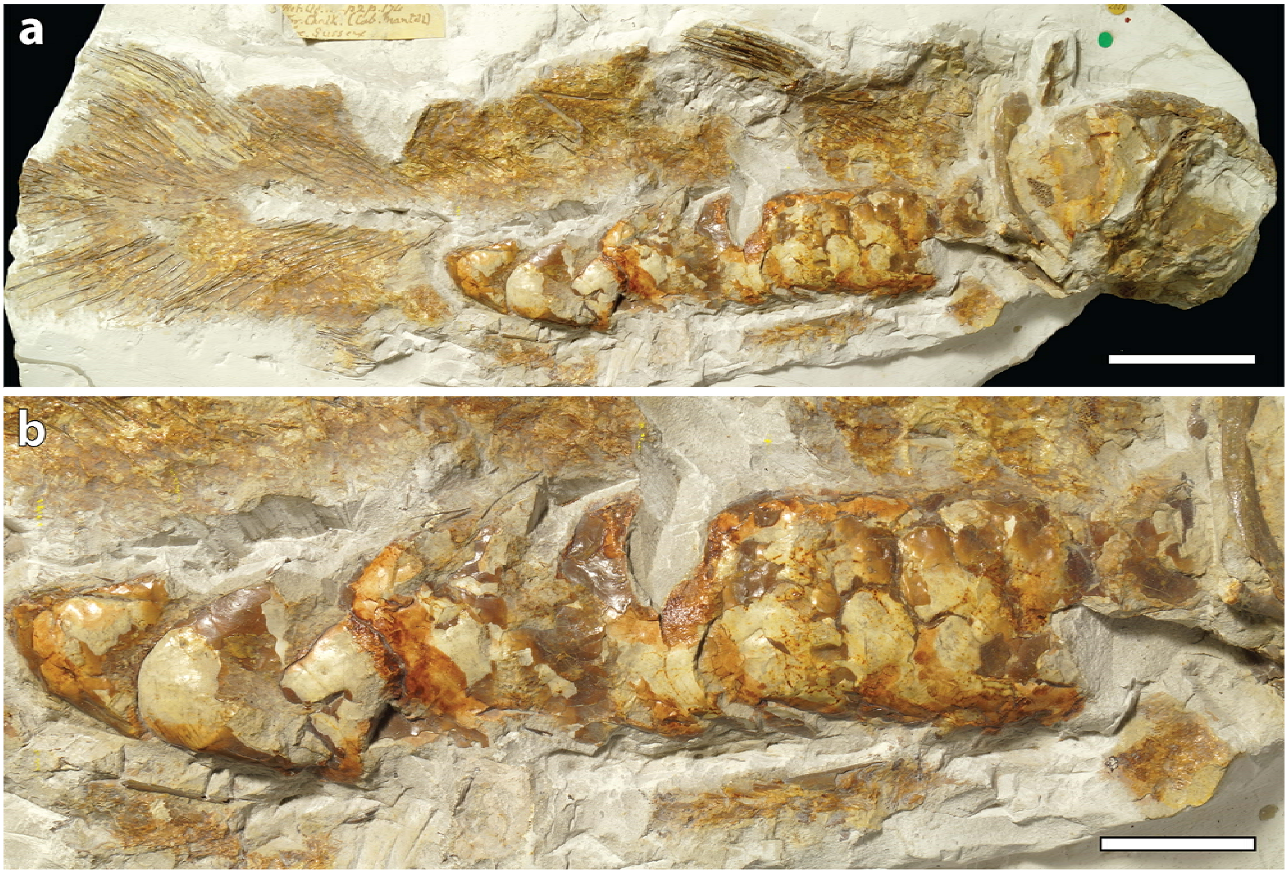

Supplementary figure 4 | Photographs of the calcified lung of the Cretaceous coelacanth *Macropoma mantelli* Agassiz, 1843 from UK, NHMUK PV P 2051. **a,** right lateral view. **b,** detail of the calcified plates. Scale bars: **a,** 5 cm; **b,** 2.5 cm.
